# Supplementary material for: A Perspective on Current Flavivirus Vaccine Development: A Brief Review
Source: Viruses. 2023 Mar 28;15(4):860. doi: 10.3390/v15040860 (PMC10142581; doi:10.3390/v15040860)
Supplement: Supplementary file 1 [file viruses-15-00860-s001.zip › viruses-2158156-supplementary.pdf]

Table S1. List of flavivirus vaccines currently being tested in clinical trials

Biological	Type of Study	Vaccine type	Phase	Trial registration number	Location
**WNV**					
WN/DEN4Δ30	Safety, Immunogenicity	Chimeric Live attenuated	1	NCT02186626	United States
WN/DEN4Δ30	Safety, Immunogenicity	Chimeric Live attenuated	1	NCT00094718	United States
WN/DEN4Δ30	Safety, Immunogenicity	Chimeric Live attenuated	1	NCT00537147	United States
HydroVax-001	Safety, Immunogenicity	Inactive	1	NCT02337868	United States
WN-80E	Safety	Subunit	1	NCT00707642	United States
VRC-WNVDNA020-00-VP	Safety, Tolerability, Immunogenicity	Genomic (DNA)	1	NCT00300417	United States
VRC-WNVDNA017-00-VP	Safety, Immunogenicity	Genomic (plasmid DNA)	1	NCT00106769	United States
ChimeriVax-WN02	Safety, Immunogenicity	Chimeric Live attenuated	2	NCT00746798	United States
**DENV**					
LD vehicle-GNP, LD PepGNP-Dengue, HD vehicle-GNP, HD PepGNP-Dengue	Safety (Co-administration)	Genomic (DNA)	1	NCT04935801	Switzerland
rDEN3Δ30	Safety, Immunogenicity	Live attenuated	1	NCT02684383	United States
TetraVax-DV TV005	Safety, Immunogenicity	Chimeric Live attenuated	1	NCT02879266	United States
TetraVax-DV-TV005, rDEN3Δ30	Safety, Efficacy (Co-administration)	Chimeric Live attenuated	1	NCT02873260	United States
TV005 vaccine	Safety, Immunogenicity	Live attenuated	2	NCT02678455	Bangladesh
TDENV-LAV, TDENV-PIV	Safety, Immunogenicity (Co-administration)	Live attenuated, Inactive	1	NCT02239614	United States
rDEN2Δ30-7169	Safety, Immunogenicity	Live attenuated	1	NCT02433652	United States
TDV	Safety, Immunogenicity	Live attenuated	3	NCT03771963	United States
TDV	Safety, Immunogenicity	Live attenuated	1	NCT01110551	United States
V180, Alhydrogel™	Safety, Tolerability, Immunogenicity	Subunit	1	NCT02450838	United States
TDV	Safety, Immunogenicity	Live attenuated	1	NCT01110551	United States
DENV-1-LVHC, TDEN-PIV	Longevity (Co-administration)	Live attenuated	1	NCT04786457	United States
DENV-1 PIV	Longevity	Inactive	1	NCT01502735	United States
TetraVax-DV-TV003	Efficacy	Chimeric Live attenuated	1	NCT03416036	United States
T-DEN F17	Safety, Immunogenicity	Live attenuated	1, 2	NCT00322049	Thailand
TDV	Safety, Immunogenicity	Live attenuated	3	NCT03341637	Mexico
TetraVax-DV	Safety, Immunogenicity	Chimeric Live attenuated	1	NCT01436422	United States
WRAIR	Longevity	Live attenuated	1	NCT00239577	United States
rDEN2/4Δ30	Safety, Immunogenicity	Live attenuated	1	NCT01073306	United States
TDENV-PIV	Safety, Immunogenicity	Inactive	1	NCT01702857	Puerto Rico
rDEN1Δ30	Safety, Immunogenicity	Live attenuated	1	NCT01084291	United States
TDENV-PIV with Alum and AS03B1 adjuvant	Safety, Immunogenicity	Inactive	1	NCT01666652	United States
D1ME100	Safety, Immunogenicity	Genomic (DNA)	1	NCT00290147	United States
rDEN1Δ30, rDEN2Δ30-7169	Longevity (Co-administration)	Live attenuated	1	NCT02392325	United States
TetraVax-DV	safety and immunogenicity	Chimeric Live attenuated	1	NCT01072786	United States
9vHPV, TDV	Safety, Immunogenicity, Longevity (Co-administration)	Inactive, Live attenuated	3	NCT04313244	Thailand
TDV	Safety, Immunogenicity, Longevity	Live attenuated	2	NCT03746015	United States

TV005, rDEN2Δ30-7169	Safety, Immunogenicity, Longevity (Co-administration)	Live attenuated	1	NCT02317900	United States
TAK-003	Longevity	Inactive	3	NCT03423173	United States
rDEN2Δ30-7169	Safety, Immunogenicity	Live attenuated	1	NCT01931176	United States
TV003	Safety, Immunogenicity, Longevity	Inactive	2	NCT02332733	Thailand
F12, JE	Safety, Immunogenicity	Live attenuated	1, 2	NCT00384670	Thailand
TetraVax-DV-TV003, rDEN2Δ30-7169	Longevity, Efficacy	Chimeric Live attenuated	1	NCT02021968	United States
rDEN3delta30/31-7164	Safety, Immunogenicity	Live attenuated	1	NCT00831012	United States
rDEN1delta30	Safety, Immunogenicity	Live attenuated	1	NCT00473135	United States
V180-001	Safety, Tolerability, Immunogenicity	Subunit	1	NCT01477580	NA
rDEN3/4delta30(ME)	Safety, Immunogenicity	Live attenuated	1	NCT00375726	United States
rDEN4delta30-4995	Safety, Immunogenicity	Live attenuated	1	NCT00322946	United States
TetraVax-DV	Safety, Immunogenicity, Longevity	Chimeric Live attenuated	1	NCT01506570	United States
HD-TDV	Safety, Immunogenicity, Longevity	Live attenuated	2	NCT02425098	Singapore
TDV IDT	Safety, Immunogenicity	Live attenuated	2	NCT02193087	United States
TVDV, Vaxfectin	Safety, Tolerability, Immunogenicity (Co-administration)	Genomic (DNA)	1	NCT01502358	United States
YF-17D, TDV	Safety, Immunogenicity, Longevity (Co-administration)	Live attenuated	3	NCT03342898	United States
rDEN4delta30-200,201	Safety, Immunogenicity (Co-administration)	Live attenuated	1	NCT00270699	United States
TDV	Safety, Immunogenicity, Longevity	Live attenuated	2	NCT02302066	Dominican Republic, Panama and Philippines
rDEN2/4delta30(ME)	Safety, Immunogenicity	Live attenuated	1	NCT00094705	United States
rDEN1delta30	Safety, Immunogenicity	Live attenuated	1	NCT00089908	United States
CYD	Safety, Immunogenicity	Chimeric Live attenuated	2	NCT01187433	Brazil
rDEN3-3'D4delta30	Safety, Immunogenicity	Live attenuated	1	NCT00712803	United States
rDEN2/4delta30(ME)	Safety, Immunogenicity	Live attenuated	1	NCT00920517	United States
T-DEN F17, T-DEN F19	Safety, Immunogenicity (Co-administration)	Live attenuated	1	NCT00370682	Thailand
CYD, DTaP-IPV//Hib	Safety, Immunogenicity, Longevity (Co-administration)	Chimeric Live attenuated, Live attenuated	3	NCT01411241	Mexico
45AZ5 PDK-27, S16803 PDK-50, CH53489 PDK-20, 341750 PDK-6	Safety, Immunogenicity, Longevity (Co-administration)	Live attenuated	2	NCT00350337	United States
CYD	Longevity	Chimeric Live attenuated	2	NCT02824198	Singapore
rDEN1delta30, rDEN2/4delta30(ME)	Safety, Immunogenicity (Co-administration)	Live attenuated	1	NCT00458120	United States
DEN vaccine F17	Safety, Immunogenicity, Longevity	Live attenuated	1, 2	NCT01843621	Thailand
CYD	Safety, Immunogenicity	Chimeric Live attenuated	1	NCT02623725	Brazil, Colombia, Honduras, Mexico And Puerto Rico
CYD	Safety, Immunogenicity, Longevity	Chimeric Live attenuated	3	NCT01254422	Malaysia
CYD	Safety, Immunogenicity, Longevity	Chimeric Live attenuated	2	NCT02628444	Colombia, Philippines
CYD	Longevity	Chimeric Live attenuated	3	NCT01134263	Australia
CYD, OKAVAX, AVAXIM	Safety, Immunogenicity (Co-administration)	Chimeric Live attenuated	2	NCT01064141	Philippines

TV003	Safety, Immunogenicity, Longevity	Live attenuated	1	NCT01782300	United States
TDENV-PIV	Safety, Immunogenicity	Inactive	1, 2	NCT02421367	United States
CYD	Longevity	Chimeric Live attenuated	2	NCT01943825	United States
TDV	Safety, Immunogenicity	Live attenuated	1	NCT01224639	Colombia
TDV	Safety, Immunogenicity	Live attenuated	1	NCT01728792	United States
CYD	Safety, Immunogenicity, Longevity	Chimeric Live attenuated	2	NCT01550289	India
CYD	Safety, Immunogenicity, Longevity	Chimeric Live attenuated	2	NCT00617344	United States
CYD	Efficacy, Safety, Longevity	Chimeric Live attenuated	3	NCT01373281	Indonesia, Malaysia, Philippines, Thailand, Vietnam
TV003	Safety, Immunogenicity	Inactive	2	NCT03485144	Taiwan
CYD	Safety, Efficacy, Longevity	Chimeric Live attenuated	3	NCT01374516	Brazil, Colombia, Honduras, Mexico, Puerto Rico
CYD	Safety, Immunogenicity, Longevity	Chimeric Live attenuated	2	NCT02993757	Malaysia
CYD	Safety, Immunogenicity	Chimeric Live attenuated	3	NCT02979535	Mexico
WRAIR	Safety, Immunogenicity, Longevity	Live attenuated	2	NCT00468858	Puerto Rico
Stamaril®, CYD	Safety, Immunogenicity, Longevity (Co-administration)	Chimeric Live attenuated, Live attenuated	3	NCT01436396	Colombia, Peru
rDEN4delta30	Safety, Immunogenicity	Live attenuated	1	NCT00919178	United States
ChimeriVax™ Dengue Tetravalent	Safety, Immunogenicity, Longevity	Chimeric Live attenuated	2	NCT00730288	Australia
CYD-1,2,3,4, VDV-2, JE-VAX®	Safety, Immunogenicity (Co-administration)	Chimeric Live attenuated	2	NCT00740155	Mexico
CYD, Verorab®	Safety, Immunogenicity, Longevity (Co-administration)	Chimeric Live attenuated, Live attenuated	2	NCT00842530	Thailand
CYD	Safety, Immunogenicity, Longevity	Chimeric Live attenuated	2	NCT00993447	Colombia, Honduras, Mexico, Puerto Rico
CYD	Safety, Immunogenicity, Longevity	Chimeric Live attenuated	2	NCT00875524	Vietnam
CYD	Safety, Immunogenicity	Chimeric Live attenuated	2	NCT00880893	Singapore
CYD, YF VAX®	Safety, Immunogenicity (Co-administration)	Chimeric Live attenuated	2	NCT00788151	Peru
HBV-001 D1	Safety	Recombinant protein	1	NCT00936429	United States
TDV	Safety, Immunogenicity	Live attenuated	1	NCT01542632	United States
Dengusiil	Safety, Immunogenicity	Live attenuated	1	NCT04035278	Australia
TDV	Safety, Immunogenicity	Live attenuated	1	NCT01542632	United States
TDV	Safety, Immunogenicity, Longevity	Live attenuated	2	NCT01511250	Colombia, Puerto Rico, Singapore, Thailand
TDV	Safety, Immunogenicity, Longevity	Live attenuated	2	NCT02948829	Panama, Philippines,
TDV	Safety, Immunogenicity, Longevity	Live attenuated	3	NCT03525119	United Kingdom
LGT(TP21)/DEN4	Safety, Immunogenicity	Chimeric Live attenuated	1	NCT00118924	United States
WN/DEN4delta30	Safety, Immunogenicity	Chimeric Live attenuated	1	NCT00537147	United States
WN/DEN4delta30	Safety, Immunogenicity	Chimeric Live attenuated	1	NCT00094718	United States
WN/DEN4delta30	Safety, Immunogenicity	Chimeric Live attenuated	1	NCT01765426	United States
**ZIKV**					
ZPIV	Safety, Immunogenicity	Inactive	1	NCT02937233	United States
ZPIV	Safety, Immunogenicity	Inactive	1	NCT02952833	United States
rZIKV/D4Δ30-713	Safety, Immunogenicity	Live attenuated	1	NCT03611946	United States
mRNA-1893	Safety, Tolerability, Immunogenicity	Genomic (mRNA)	1	NCT04064905	United States, Puerto Rico

MV-ZIKA-RSP	Safety, Immunogenicity	Viral vector-based	1	NCT04033068	Austria
MV-ZIKA-RSP	Safety, Immunogenicity	Viral vector-based	1	NCT02996890	Austria
ZPIV	Safety, Immunogenicity	Inactive	1	NCT03008122	Puerto Rico
ZPIV with aluminum hydroxide adjuvant.	Safety, Reactogenicity, Immunogenicity	Inactive	1	NCT02963909	United States
VRC-ZKADNA085-00-VP	Safety, Immunogenicity	Genomic (DNA)	1	NCT02840487	United States
VRC-ZKADNA090-00-VP	Safety, Immunogenicity	Genomic (DNA)	1	NCT02996461	United States
VLA1601	Safety, Immunogenicity	Inactive	1	NCT03425149	United States
mRNA-1325	Safety, Tolerability, Immunogenicity	Genomic (mRNA)	1	NCT03014089	United States
ChAdOx1 Zika	Safety, Immunogenicity	Subunit	1	NCT04015648	United Kingdom
BBV121	Safety, Immunogenicity	Inactive	1	NCT04478656	India
VRC-ZKADNA090-00-VP	Safety, Immunogenicity, Efficacy	Genomic (DNA)	2	NCT03110770	United States
GLS-5700	Safety, Tolerability, Immunogenicity	Genomic (DNA)	1	NCT02887482	Puerto Rico
GLS-5700	Safety, Tolerability, Immunogenicity	Genomic (DNA)	1	NCT02809443	United States, Canada
**YFV**					
XRX-001	Safety, Tolerability, Immunogenicity	Inactivated	1	NCT00995865	United States
YFV-17D	Immunogenicity	Live attenuated		NCT00616356	United States
YEFE	Safety, Immunogenicity	Live attenuated	4	NCT02991495	Kenya and Uganda
Stamaril	Immunogenicity	Live attenuated	3	NCT01426243	France
ChimeriVax™-JE, Stamaril	Safety, Immunogenicity (Co-administration)	Chimeric Live attenuated	2	NCT00982137	Australia
Stamaril	Immunogenicity	Live attenuated	2	NCT03116802	Singapore
CYD, Stamaril®, MMR, Pneumococcal Conjugated Vaccine, Hepatitis A Pediatric Vaccine, DTaP IPV//Hib Vaccine	Safety, Immunogenicity (Co-administration)	Chimeric Live attenuated, Live attenuated	3	NCT01436396	Colombia and Peru
IXIARO, Stamaril®	Efficacy (Co-administration)	Inactive and Live attenuated	2	NCT01943305	Singapore
17D YF, YF-VAX®	Safety, Immunogenicity (Co-administration)	Live attenuated	1	NCT00254826	United States
MMR/YF	Immunogenicity	Live attenuated	4	NCT03368495	Argentina
YF VAX®, CYD Dengue Vaccine	Immunogenicity (Co-administration)	Chimeric Live attenuated	2	NCT01488890	United States
YF-17D, DENVax	Immunogenicity, Safety (Co-administration)	Live attenuated	3	NCT03342898	United States
YFV-17D	Immunogenicity	Live attenuated	2	NCT00723489	United States
Stamaril, SB257049, MR-Vac, Vitamin A	Safety, Immunogenicity (Co-administration)	Live attenuated	3	NCT02699099	Ghana
GSK 257049, Tritanrix™ HepB/Hib, Rouvax™, Stamaril™, Polio Sabin™	Safety, Immunogenicity (Co-administration)	Live attenuated	2	NCT00436007	Gabon, Ghana and Tanzania
**TBEV**					
TBE vaccine	Safety, Immunogenicity, Tolerability	Not available	3	NCT04648241	Japan

Encepur, FSME-IMMUN	Longevity (Co-administration)	Inactive	4	NCT01106482	Germany
LGT(TP21)/DEN4	Safety, Immunogenicity	Live attenuated	1	NCT00118924	United States
TicoVac	Longevity	Inactive	4	NCT01710189	United Kingdom
TBEvac	Longevity	Inactive	4	NCT01562444	Czechia
FSME-immune	Longevity	Inactive	2, 3	NCT02318069	Finland, Sweden
FSME-Immun	Longevity	Inactive	4	NCT00804219	Austria
FSME Immun	Longevity	Inactive	2	NCT01991067	Austria
FSME-IMMUN	Longevity	Inactive	4	NCT01582698	Poland
FSME-IMMUN	Safety	Inactive	3	NCT00161863	Austria, Germany, Poland
FSME-IMMUN	Safety, Immunogenicity	Inactive	2	NCT00161798	Germany
FSME-IMMUN	Longevity	Inactive	4	NCT00161785	Poland
FSME IMMUN New	Safety, Immunogenicity	Inactive	2	NCT00161772	Austria, Germany
FSME-IMMUN	Longevity	Inactive	4	NCT00894686	Austria, Germany, Poland
FSME-IMMUN	Longevity	Inactive	4	NCT02511535	Austria
FSME-IMMUN, Encepur	Safety, Immunogenicity (Co-administration)	Inactive	3	NCT00840801	Austria, Czech Republic
FSME-IMMUN	Longevity	Inactive	4	NCT00503529	Poland
Encepur Adults	Longevity	Inactive	4	NCT03294135	Czechia
FSME Immun CC	Longevity	Inactive	4	NCT00461695	Switzerland
FSME-IMMUN, TicoVac	Longevity (Co-administration)	Inactive	4	NCT00163618	Austria
FSME Immun	Longevity	Inactive	4	NCT00163540	Belgium
FSME Immun	Safety, Immunogenicity	Inactive	4	NCT00161954	Belgium
FSME Immun	Longevity	Inactive	3	NCT00161876	Poland
TICOVAC	Safety, Immunogenicity	Inactive	2, 3	NCT00161746	Austria
FSME Immun	Longevity	Inactive	3	NCT00460486	Poland
FSME Immun	Longevity	Inactive	2	NCT00161889	Germany
FSME Immun	Longevity	Inactive	2	NCT00161850	Austria and Germany
FSME IMMUN New, ENCEPUR	Safety, Tolerability (Co-administration)	Inactive	3	NCT00161824	Poland
**JEV**					
JECEVAX	Safety	Inactive	2	NCT03204227	Vietnam
IMOJEV®	Safety, Immunogenicity	Chimeric live attenuated	3	NCT02492165	Vietnam
IMOJEV®	Safety, Immunogenicity	Chimeric live attenuated	3	NCT01900444	Korea
IMOJEV®	Safety, Immunogenicity, Longevity	Chimeric live attenuated	4	NCT01981967	Thailand
IXIARO (IC51)	Safety, Immunogenicity, Longevity	Inactive	4	NCT01158599	Austria, Germany
IXIARO	Longevity	Inactive	3	NCT01296360	Philippines
SA14-14-2	Longevity	Live attenuated	4	NCT01656200	India
IMOJEV™, SA14-14-2	Safety, Immunogenicity (Co-administration)	Live attenuated	3	NCT01396512	Korea
SA 14-14-2	Safety, Immunogenicity	Live attenuated	4	NCT01567865	Bangladesh
IC51, JE-VAX	Safety, Immunogenicity (Co-administration)	Inactive	3	NCT00595465	Austria, Germany
SA 14-14-2	Safety, Immunogenicity, Longevity	Live attenuated	4	NCT00463684	Sri Lanka,
SA 14-14-2	Longevity	Live attenuated	3	NCT00412516	Philippines
SA 14-14-2	Safety, Immunogenicity	Live attenuated	3	NCT01047839	Australia, United States, Denmark, Germany, Sweden
SA 14-14-2	Longevity	Live attenuated	4	NCT01635816	Bangladesh
SA 14-14-2	Safety, Immunogenicity, Longevity	Live attenuated	4	NCT00463476	Sri Lanka

SA 14-14-2	Safety, Tolerability, Immunogenicity	Live attenuated	2	NCT00319592	United States
ChimeriVax™-JE, JE-VAX	Safety, Immunogenicity, Tolerability (Co-administration)	Chimeric live attenuated, Inactive	2, 3	NCT00314145	United States, Australia
ChimeriVax™-JE	Safety, Tolerability	Chimeric live attenuated	3	NCT00735644	Philippines, Thailand
JE-CV, Varicella	Safety, Immunogenicity, Longevity (Co-administration)	Chimeric live attenuated	3	NCT01190228	Philippines
JE-CV, SA14-14-2	Safety, Immunogenicity (Co-administration)	Chimeric live attenuated, Live attenuated	3	NCT01092507	Thailand
ChimeriVax™-JE	Safety, Tolerability, Immunogenicity	Chimeric live attenuated	2	NCT00621764	Thailand
ChimeriVax™-JE	Safety, Tolerability, Immunogenicity	Chimeric live attenuated	2	NCT00981630	Australia
ChimeriVax™-JE	Safety, Tolerability, Immunogenicity	Chimeric live attenuated	3	NCT00314132	United States, Australia
ChimeriVax™-JE	Safety, Immunogenicity, Longevity	Chimeric live attenuated	2	NCT00981175	Australia
ChimeriVax™-JE, JE-MBDV	Safety, Tolerability, Immunogenicity (Co-administration)	Chimeric live attenuated	2	NCT00441259	India
Different doses of JEVAX	Safety, Immunogenicity	Inactive	2	NCT02816554	Vietnam
Different doses of JEVAX	Safety, Immunogenicity	Inactive	3	NCT03282370	Vietnam
BR JEV, KD-287 ENCEVAC or JEV-GCC	Longevity (Co-administration)	Inactive	4	NCT02532569	Korea
KD-287, JE vaccine	Safety, Immunogenicity (Co-administration)	Inactive	3	NCT01150942	Korea
IC51, HARVIX	Safety, Immunogenicity (Co-administration)	Inactive and	3	NCT00596271	Philippines
IC51, HARVIX, Prevnar	Safety, Immunogenicity (Co-administration)	Inactive and	3	NCT01041573	Philippines
IC51(IXIARO®, JESPECT®)	Safety, Immunogenicity, Longevity	Inactive	3	NCT01246479	United States, Australia, Germany
IC51	Immunogenicity and Longevity	Inactive	3	NCT00776230	Australia, Germany
LJEV, MCV	Immunogenicity (Co-administration)	Live attenuated	4	NCT02643433	China
JE-CV	Safety, Immunogenicity, Longevity	Chimeric Live attenuated	3	NCT01001988	Philippines and Thailand
JE-CV, MMR	Immunogenicity (Co-administration)	Chimeric live attenuated, Live attenuated	3	NCT01188343	Taiwan
Chimeravax™-JE, Stamaril®	Safety, Tolerability, Immunogenicity (co-administration)	Chimeric live attenuated, Live attenuated	2	NCT00982137	Australia
SA 14-14-2, MV	Safety, Immunogenicity (co-administration)	Live attenuated	3	NCT00249769	Philippines
JEVAC	Safety, Immunogenicity, Longevity	Inactive	3	NCT01408537	Thailand
SA 14-14-2, MMR	Safety, Immunogenicity (Co-administration)	Live attenuated	4	NCT02880865	Philippines
IMOJEV, CD JEVAX	Safety, Immunogenicity (Co-administration)	Chimeric, Live attenuated	4	NCT02526550	Thailand
